# Milk fat globule membrane promotes brain development in piglets by enhancing the connection of white matter fiber trace

**DOI:** 10.3389/fnut.2023.1248809

**Published:** 2023-11-23

**Authors:** Yingqian Zhang, Bangcheng Zhao, Szeto Ignatius Man-Yau, Zhixiang Pan, Lijuan Gao, Qinxi Li, Cheng Tang, Yu Wang, Xun Tang, Zifu Zhao, Jingyu Hao, Sufang Duan, Yalu Yan, Ting Li, Zhihui Zhong

**Affiliations:** ^1^Laboratory of Nonhuman Primate Disease Modeling Research, Department of Neurology, West China Hospital, Sichuan University, Chengdu, China; ^2^Inner Mongolia Dairy Technology Research Institute Co. Ltd., Yili Innovation Center, Inner Mongolia Yili Industrial Group Co., Ltd., Hohhot, China; ^3^Department of Radiography, West China Hospital, Sichuan University, Chengdu, China

**Keywords:** MFGM, memory-improvement, fractional anisotropy (FA), BDNF, infant diet

## Abstract

**Introduction:**

Brain development during infancy is crucial for later health and development. Although Milk Fat Globule Membrane (MFGM) has been demonstrated to enhance brain development, further investigation is needed to determine the optimal dose.

**Methods:**

In this study, 80 piglets aged 2 days were randomly assigned to four groups: Control group, MFGM-L (1.74 g MFGM per 100 g diet), MFGM-M (4.64 g MFGM per 100 g diet), and MFGM-H (6.09 g MFGM per 100 g diet). Daily body weight and milk intake of the piglets were recorded until 31 days postnatal. Learning and memory abilities were evaluated using the spatial T-maze test on day 15. MRI analysis was conducted to assess functional and structural changes in brain tissues. Additionally, mRNA and protein expression of brain-derived neurotrophic factor (BDNF) and neurotrophin-3 (NTF-3) in the hippocampus and prefrontal cortex were evaluated.

**Results:**

The results indicated that the MFGM supplemented diet significantly improved the accuracy of the piglets in the T-maze test, with the MFGM-L group exhibiting the best performance. MRI showed no volumetric differences in the gray and white matter between the groups. However, the fractional anisotropy in the left and right hippocampus of piglets in the MFGM-L group was significantly higher than in the other three groups. Furthermore, there was a strong correlation between the accuracy of the T-maze test and hippocampal fractional anisotropy.

**Discussion:**

The MFGM supplemented diet also increased the expression of BDNF in the cerebral cortex. However, the changes in BDNF were not consistent with the results of the T-maze test. In conclusion, adding 1.74 g MFGM per 100 g diet can significantly improve neonatal piglets’ learning and memory abilities, potentially by enhancing the connection of white matter fiber bundles in the brain.

## 1 Introduction

The human brain develops rapidly in the last weeks of gestation and the first 2 years of life; brain development is highly dynamic during the fetal stage and the first 2 years after birth (1). Although WHO recommends exclusive breastfeeding up to 6 months of age, 55.8% of infants need formula feeds (2) when breastfeeding is not possible, suitable, or adequate, such as lack of breast milk, maternal and infant disease, and maternal separation (3). There was reported poorer cognitive development in formula-fed infants than in breastfed infants (4). Compared with the formula, these differences may be partially associated with the high concentration of phospholipids, sphingolipids, and ganglioside in the breast formula (5). There is a need to develop formula milk that closely mimics the benefits of breast milk (6).

The milk fat globule membrane (MFGM) has the structure of lipids surrounding every fat globule in breast milk (7). It contributes to 0.2–2% of total fat in breast milk, is the primary source of phospholipids, and plays an essential role in fat delivery (8). The MFGM added to infant formula was demonstrated to be safe (9), and it had potential benefits for brain development (10), such as improving spatial learning (11), promoting reflex development (12), and increasing hippocampal expression of genes related to neurodevelopment (13). However, the production cost of MFGM is relatively high due to its unique composition, which can result in higher prices for MFGM-enriched products.

To achieve a balance between efficacy and affordability, it is crucial to determine the most effective formulation of MFGM that can provide significant functional benefits while falling within a reasonable price range. Additionally, optimizing the composition of MFGM may have environmental benefits, such as reducing food waste and minimizing resource depletion (14). Thus, studying the optimal blend of MFGM is vital for striking a balance between functionality and pricing, which has the potential to benefit both consumers and the environment (15). In addition, the mechanisms underlying optimal MFGM proportion have yet to be fully explored.

Therefore, the purpose of this study is to test the learning and memory, as well as brain development, in neonatal piglets, fed a nutritionally complete diet of the MFGM supplemented diet of three different concentrations. As a large and gyrencephalic species, there are striking similarities between piglets and humans, making the piglet the ideal model for pediatric nutrition and metabolism research (16). The hypothesis was that The brain development of piglets varied depending on the dosage of the MFGM diet. One certain concentration of MFGM-enriched diet would promote brain growth and improve learning and memory.

## 2 Materials and methods

### 2.1 Animals and housing

**Figure 1:**
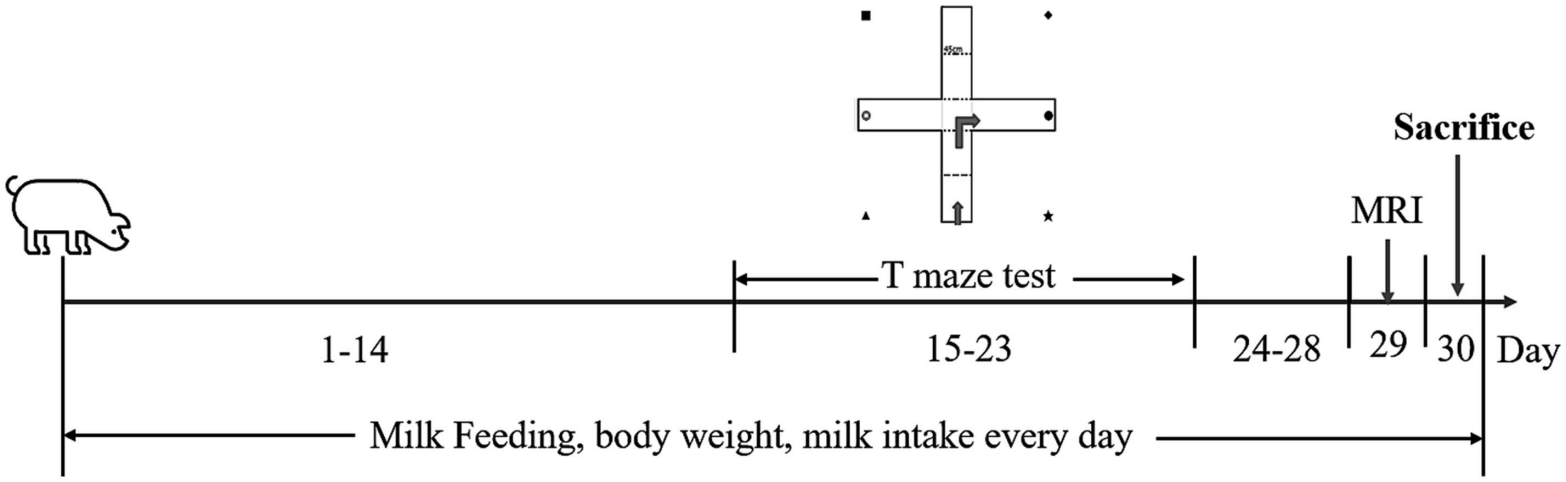
Timeline of the experimental procedure. Piglets aged 2 days postnatal (day 1) were brought to the animal facility. They were fed with a nutritionally complete, customized milk replacer for a month. The T-maze tests were performed from day 15 to 23 for spatial learning and memory assessment. MRI was conducted on day 29. The piglets were all sacrificed on day 30.

Eighty male ternary hybrid piglets (Landrace × Yorkshire × Duroc, 2 days postnatal, 1.3–2.0 kg each) used in this study were provided by Chengdu Shepherd Boy Village Agricultural Development Co., Ltd., Sichuan Province, China. Two piglets were excluded because of poor growth state. Due to the facility space limitations, the animal experiments were conducted in five separate sessions, each consisting of 16 piglets obtained from three to six different sows. All animals were housed and kept in separate cages at the KangCheng facility [Sichuan KangCheng Biotech Co., Ltd.; animal production license number: SYXK (Chuan) 2019–215] in pig cages for 30 days under controlled temperature: 22 ± 2°C, humidity: 60% ~ 70% under a 12 h-alternate light/dark cycle (lights on at 8:00 am and off at 8:00 pm). Fresh milk powder was provided to the piglets every 2 h a day. At the facility, the standard room temperature was set at 24.5°C. Initially, the temperature in the cage was higher than the standard temperature (33–35°C). Then, the piglets were housed in cages with decreased temperatures as they grew. If a piglet developed diarrhea, it was given a saline rehydration solution. At the facility, 16 two-day-old newborn male piglets per entry were randomly divided into four groups according to body weight when piglets entered the facility, four piglets for each group in every session, and the piglets were reared until 31 days postpartum (Figure 1). All experiments followed the guidelines for the Care and Use of Laboratory Animals provided by the Ministry of Science and Technology of the People’s Republic of China (PRC). All animal care and experimental procedures were performed according to the Animal Research: Reporting *in Vivo* Experiments (ARRIVE) guidelines (17) and were approved by the Institutional Animal Care and Use Committee (IACUC) of West China Hospital Sichuan University. The environmental breeding conditions followed the PRC national standard GB14925-2010.

### 2.2 Dietary

In this study, all artificially reared piglets were fed milk powder provided by Inner Mongolia Dairy Technology Research Institute Co., Ltd. Piglets in the control group (*n* = 19) were fed the control diet, the other groups were provided with diet supplemented with milk fat globule membrane (MFGM; Lacprodan^®^ MFGM-10, Arla Foods Ingredients) of 1.74 g/100 g MFGM (MFGM-L, *n* = 19), 4.64 g/100 g MFGM (MFGM-M, *n* = 20), and 6.09 g/100 g MFGM (MFGM-H, *n* = 20). The specific diet in each group are listed in Table 1. The difference in the four groups mainly comes from the concentration of total phospholipids, phosphatidyl serine, phosphatidyl ethanolamine, phosphatidyl inositol, and phosphatidylcholine, as indicated by the bold part in Table 1. According to the nutritional needs of piglets (nutritional requirement criterion, NRC 2022), the amount of piglets’ daily dietary intake is 285 mL/kg*fasting body weight + 50 mL. The milk was freshly prepared with warm water (37–40°C) every 4–5 h, in a 1:4 (powder-to-water) ratio, and the prepared milk was placed in a feeder.

**Table 1:** The nutritional diet in each group.

	Control	MFGM-L	MFGM-M	MFGM-H
Energy KJ/100 g	464	466	465	464
Protein, g/100 g	23.8	23.6	23.5	23.6
Fat, g/100 g	21.3	21.7	21.4	21.3
Calcium, mg/kg	14,000	14,000	14,000	15,000
Phosphate, mg/kg	846	855	880	880
Zinc, mg/kg	88.8	87.5	87.8	89.3
VE mg/100 g	113	115	113	111
Total phospholipids, g/100 g	0.28	0.36	0.48	0.59
Phosphatidyl serine, g/100 g	0.03	0.04	0.06	0.08
Phosphatidyl ethanolamine, g/100 g	0.07	0.09	0.12	0.14
Phosphatidyl inositol, g/100 g	0.02	0.03	0.04	0.04
Phosphatidylcholine, g/100 g	0.08	0.1	0.13	0.16
Sphingomyelin, g/100 g	0.08	0.1	0.13	0.17

The piglets’ daily dietary intake is 285 mL/kg*body weight + 50 mL (fasting weight, ranging from 1.7 to 10 kg). The daily dietary intake of a 1.5 kg piglet is 285 mL/kg*1.5 kg + 50 mL = 477.5 mL, which equals 0.2 g/mL = 95.5 g; MFGM intake is 95.5 g*1.74 g/100 g = 1.66 g. The recommended milk volume for infants aged 0–6 months is 750 mL/day. The corresponding MFGM intake is equivalent to 1.66 g*750 mL/477.5 mL = 2.60 g in infants.

### 2.3 Spatial T-maze test

Spatial learning and memory were assessed by the spatial T-maze test, the task first used to compare the performance of different pigs by Mendl et al. in 1997 (18). The behavioral device comprises a 45 cm wide cross maze with a removable acrylic clapboard. Piglets can identify the orientation from all four corners using visual cues. Two bowls were placed at T-maze’s left (west) and right (east) arms, one with milk as a reward and another empty (Supplementary Figure 1A). Topscan software (Cleversys Co. Ltd. United States version 8, 2020) assessed the piglet’s motion trails. The nine-day T-maze behavioral test included a six-day acquisition and three-day reversal phases (19). On the morning of day 15, the piglets were fasted (a nightly 6-h food deprivation period), removed from their home cages, and carried to the maze in the adjacent behavioral room until the behavior assessments were completed. In the acquisition phase, they were randomly placed at the northern or southern entrance of the maze (Supplementary Figure 1). They aimed to find the reward milk bowl via visual cues in 60 s. Each piglet was assessed 10 times daily for 6 days between 0900 and 1700 by the same two trained experimenters, and the correct rate was recorded. Within the 3-day reversal phase, the reward milk bowl and visual cues were swapped to assess the piglets’ memory and to ensure that the piglets’ orientation discernment was not self-centered. The number of incomplete/completed tasks within 60 s and the correct latency were also recorded. All behavioral technicians were blinded to the grouping information of the intervention dietary. The test indices collected include the correct rate (piglets choose reward milk bowl to represent correct), qualification (correct rate reaching 80%), non-qualified rate (the whole rate of animal in correct rate not reaching 80% across the entire test), and the earliest qualified day (the first-day piglets achieving qualification). The trace of pigs in the T-maze was shown in Supplementary Video.

### 2.4 Magnetic resonance imaging

#### 2.4.1 Data collection

MRI was conducted on day 29 using SIGNA^™^ Architect AIR^™^ Edition 3.0 Tesla, 70 cm MRI scanner (GE, United States) with a 64-channel head coil to evaluate the piglet’s brain development. Piglets were anesthetized by intramuscular injection of 0.1 mg/kg Zoletil 50 (Virbac, France). Vital signs were monitored after anesthesia. The 3D T1-weighted image (3D-T1) and diffusion tensor imaging (DTI) were scanned sequentially.

The parameters of 3D-T1 scanning were as follows: 0.7 mm thick; Frequency coding direction: front and back; Repetition time 9.1 s; Echo time 3.6 s; Layer number: 100; Signal-to-noise ratio 100%; Matrix: 256 × 256, excitation times: 2; Flip Angle: 12; The bandwidth: 31.25; Scan time: 6:29.

The procedure setting of DTI scanning is as follows: slice thickness 2 mm; Frequency coding direction: right–left; Repetition time: 6826 s; Echo time: 122.3 s; Slices: 26; Signal-to-noise ratio 70%; Matrix:128 × 128, T2 excitation times: 1; The bandwidth: 166.7; B value: 1000; Diffusion direction: 32; Scanning time: 3:52.

#### 2.4.2 Postprocessing

3D-T1 Post-imaging analysis with GE imaging workstation (GE, United States) generated axial, coronal, and sagittal images to calculate the gray and white matter volume. Then, the post-imaging analysis of piglet images, previous DTI, and voxel-based morphological (VBM) measurements was also conducted. Based on DTI, the fractional anisotropy (FA) of four regions of interest (ROIs), hippocampus (left and right, respectively), corpus callosum, and whole brain, were calculated by MRI postprocessing station. The gradient echo T1 MRI file was a 2D tomography file, which was converted into a 3D file for Voxel-based morphological processing using the Neuroimaging Informatics Technology Initiative. Brain tissue was segmented based on the Trusted Platform Module in the Statistical Parametric Mapping software (version 12); 3D files spatially distributed images of gray matter and white matter were obtained by smooth processing. Finally, voxel-based statistics were performed using the images of gray matter and white matter.

### 2.5 Quantitative RT-PCR

All the piglets were anesthetized by intramuscular injection of Zoletil 50 (Vibrac, French, 0.2 mg/kg). The brains were dissected and weighed. The hippocampus and prefrontal cortex (PFC) were excised from the fresh brain tissue and frozen in liquid nitrogen. Total RNA was isolated from 40 to 100 mg of hippocampal and prefrontal cortex tissues. Tissues were dissolved with 1 mL Trizol (Beyotime Biotechnology) and purified with organic solvent (Chloroform, isopropyl alcohol, and alcohol). The isolated total RNA was then diluted to 500 ng/μL. RNA was converted into cDNA with a reverse transcription kit (Vazyme Biotech Co., Ltd., R323-01). BDNF content was measured with a qPCR kit (Vazyme Biotech Co., Ltd., Q711-02). The purity and concentration of the RNA were evaluated by spectrophotometry (Thermo-Nano Drop 2000c-spectrophotometer) by measuring the mean absorbance ratio and optical density of all RNA at 260/280 nm. BDNF and β-actin primer sequences were as follows: BDNF primer sequence (F: 5’-AGCATTAGCGAGTGGGTGAC-3′; R: 5’-GGGACTTTTTCGAGGACCGT-3′; β-actin primer sequence: F: 5’-GACTGCGCCCATAAAACCC-3′, R: 5’-CACGAGCGCCAGCAATATCGT-3′). The qPCR parameters were as follows: pre-denaturation at 95°C, 30 min, 40 cycles reaction procedure: denaturation 95°C, 10 s; Annealing 55°C, 30 s; The fusion curve program was 95°C 10 s, 55°C 30 s, and 95°C 10 s. (The relative quantification of the transcription levels of the copy melting curve genes was performed by the ^-ΔΔ^CT method).

### 2.6 Western blot

We weighed 40 mg of porcine hippocampal tissue and lysed in 1 mL buffer Radio Immunoprecipitation Assay (RIPA; Beyotime, P0013B) with 10 μL phenylmethylsulfonyl fluoride (PMSF; Servicebio, G2008); added 400 μL RIPA for lysis tissue (the RIPA concentration was 10 μL/mg). The tissue lysates were homogenized and lysed on ice for 20 min and centrifuged at 15000 g for 20 min at 4°C. The supernatant was collected as a total protein extract. BCA Protein Detection Kit (Beyotime, P0010S) measured the protein concentration. The total protein was added to 1 × SDS-loading buffer and boiled for 5 min at 100°C. Thirty microgram protein was loaded on 12% SDS polyacrylamide gel (GenScript, M01210C). The separated protein was transferred to the polyvinylidene fluoride (PVDF) membrane (Beyotime, FFP32) and washed with Tris Buffered saline Tween (TBST). At room temperature, the membrane was blocked in a 5% nonfat milk powder blocking buffer for 2 h. The membrane was then incubated with primary antibody [Anti-BDNF antibody (Bioss, bs-4989R, 13 kDa, China); neurotrophin-3 (NTF3) antibody (Affinity, DF7235, 31 kDa); Rabbit anti-beta-actin antibody (Bioss, bs-0061R, 42 kDa)] at 4°C overnight followed by the incubation with secondary antibody HRP-conjugated affinipure goat anti-rabbit IgG (Proteintech, SA00001-2) 1:5000 for 2 h at room temperature. For imaging, they were treated with a chemiluminescence detection kit (Millipore, P90719) and exposed to a chemistry luminescence imaging system (Fluor chem. Co. model protein simple FC3, United States). Each band was quantified using ImageJ software (version 8).

### 2.7 Statistical analysis

In this study, all data were normally distributed and presented as Mean ± Standard Error of the Mean (SEM). GraphPad Prism 8 software (version 3.1) was used to plot the data. The data were analyzed by SPSS 22.0 statistical software (IBM Corporation, version 26). The parameters were evaluated for variance homogeneity by the Kolmogorov–Smirnov test. Repeated measures analysis of variance (ANOVA) was performed to determine the main effects and interaction effects of body weight, food intake, correct rate, correct latency, and proportion correct at different time points. Differences among the single index, such as white matter volume and FA, were analyzed by one-way ANOVA, followed by Bonferroni *post-hoc* tests. The correlations between correct rate and FA in different regions were analyzed statistically by Pearson’s correlation. A *p* < 0.05 was considered statistically significant.

## 3 Results

### 3.1 Dietary MFGM was well-tolerated and supported growth of piglets in 31 days

In this study, the piglets were fed with a diet containing different percentages of MFGM. In 30 days, the body weight of piglets was not affected (Supplementary Figure 2A); all body weight gain remained at normal levels of growth [*F*_(3, 74)_ = 0.295, *p* = 0.829, Supplementary Figure 2B]. Milk intake showed no difference between groups and stayed at average increases as the piglets’ development changed [Supplementary Figure 2C; F_(3, 74)_ = 0.348, *p* = 0.790, Supplementary Figure 2D]. As part of the total body weight, the brain weight also showed no difference between the groups. These results indicated that dietary MFGM was well-tolerated for piglets and supported growth. Besides, it was reported that formula feeding increased the risk of diarrhea (20). Unfortunately, two piglets had to be eliminated from the study due to the severity of their diarrhea. For the remaining piglets, the incidence of diarrhea was not alarmingly high. On average, about two or three piglets would experience bouts of diarrhea during each round of experimentation. Notably, this was particularly prevalent within the first 10 days after birth. It is worth mentioning that there were no discernible differences in the incidence of diarrhea among the various groups studied. Overall, dietary MFGM was well-tolerated and supported piglet growth and milk intake in 31 days.

### 3.2 MFGM improved piglet performance in the T-maze task

**Figure 2:**
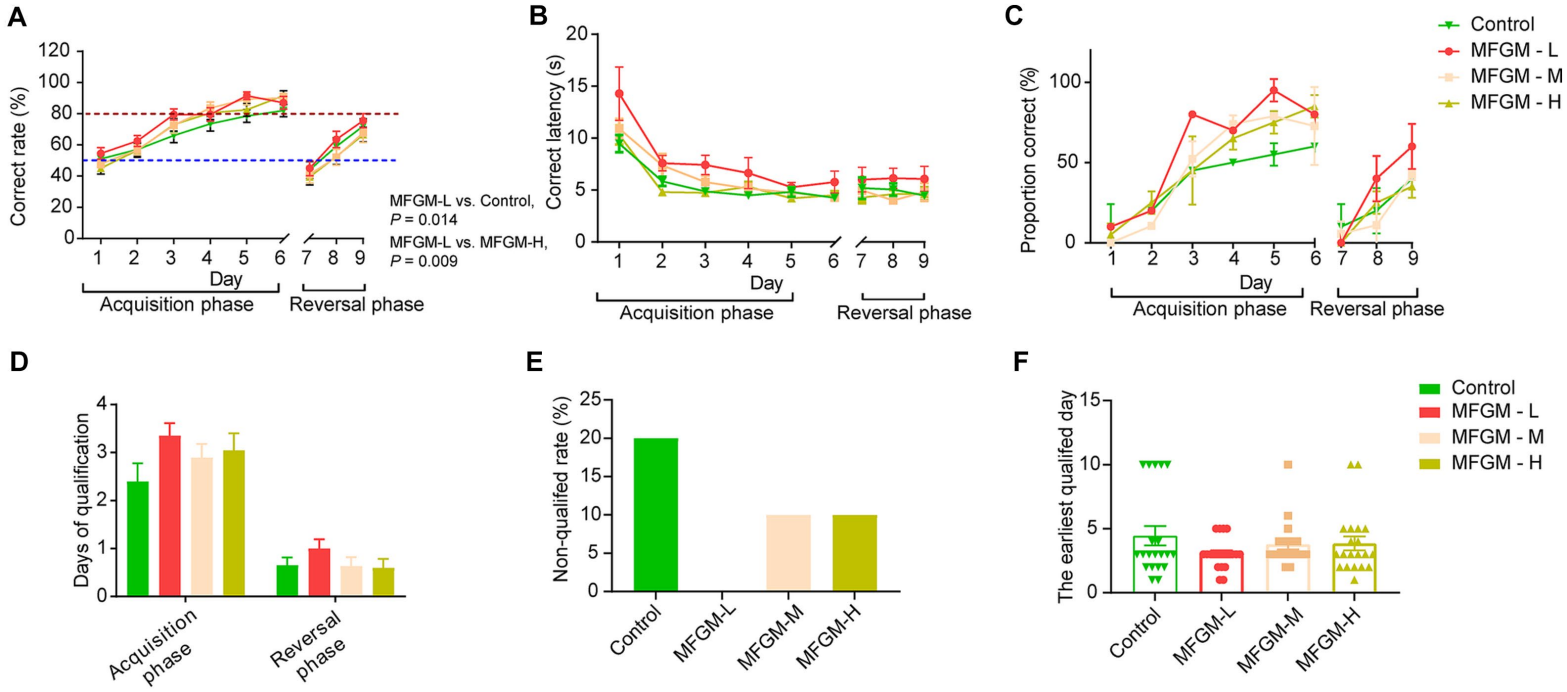
T-maze performance of piglets aged 16–24 days. Correct rate **(A)**; Correct latency **(B)**; Proportion correct rate **(C)**; Days of qualification **(D)**; Non-qualified rate **(E)**; The earliest qualified days **(F)**. *n* = 20 in Control and MFGM-H groups; *n* = 19 in MFGM-L and MFGM-M groups. Data were presented as mean ± SEM.

The spatial T-maze test was performed on day 15 of feeding, and the trial was divided into two phases: the acquisition and reversal phases. The difference between the acquisition and reversal phases was the direction of visual cues (Supplementary Figure 1). The acquisition phase lasted 6 days, while the reversal phase lasted 3 days. The most important indicator of the T-maze is the correct rate in the 10 trials every day. During the acquisition phase, the correct rate of the piglets improved steadily over time. They completed the learning process by day 3, defined as achieving an accuracy rate of 80%. By day 5, most of the tested piglets met the specified criterion. On day 7, when the reversal phase started, the accuracy rate dropped sharply due to the changed position of the visual cues. Although the accuracy rate improved on the following days (8 and 9), it did not return to the comparable performance level achieved during the acquisition phase (Figure 2A). Repeated measures revealed a day effect on correct rate (*F*_(8, 68)_ = 10.822, *p* < 0.001) and no interaction effects between days and groups [*F*_(24, 210)_ = 0.889, *p* = 0.617]. Difference existed among four group [*F*_(3, 75)_ = 3.049, *p* = 0.034]. The *post-hoc* Bonferroni analysis showed that the MFGM-L group had the highest correct rate, much higher than the control (*p* = 0.011) and MFGM-H groups (*p* = 0.044; Figure 2A). There was a day effect of latency to choice [F_(8, 68)_ = 10.822, *p* < 0.001] and no interaction effects between days and groups. Significant differences in latency to make a choice existed in each group [F_(3, 75)_ = 3.049, *p* = 0.034]. However, the *post-hoc* Bonferroni analysis showed only a trend of difference between Control and MFGM-L (*p* = 0.083), as well as MFGM-L and MFGM-H (*p* = 0.054; Figure 2B). The correct proportion demonstrated that in neither the acquisition nor the reversal phase, the MFGM-L had the highest proportion correct (Figure 2C).

The qualification days of the MFGM-L group also showed the best results in the T-maze test’s acquisition and reversal phases (Figure 2D). In the MFGM-L group, all piglets were qualified (achieving a correct rate of 80%) in both the acquisition and reversal phases. However, 20% of piglets were non-qualified in 9 days. The other two groups had the same non-qualified rates, higher than MFGM-L but lower than the control (Figure 2E). The other index of T-maze was the earliest qualified day; piglets in the MFGM-L group learned faster than the other groups, but no difference in learning rate was identified among the four groups [*F*_(3, 75)_ = 1.199, *p* = 0.316; Figure 2F].

### 3.3 MFGM promoted the growth of white matter fiber bundles

**Figure 3:**
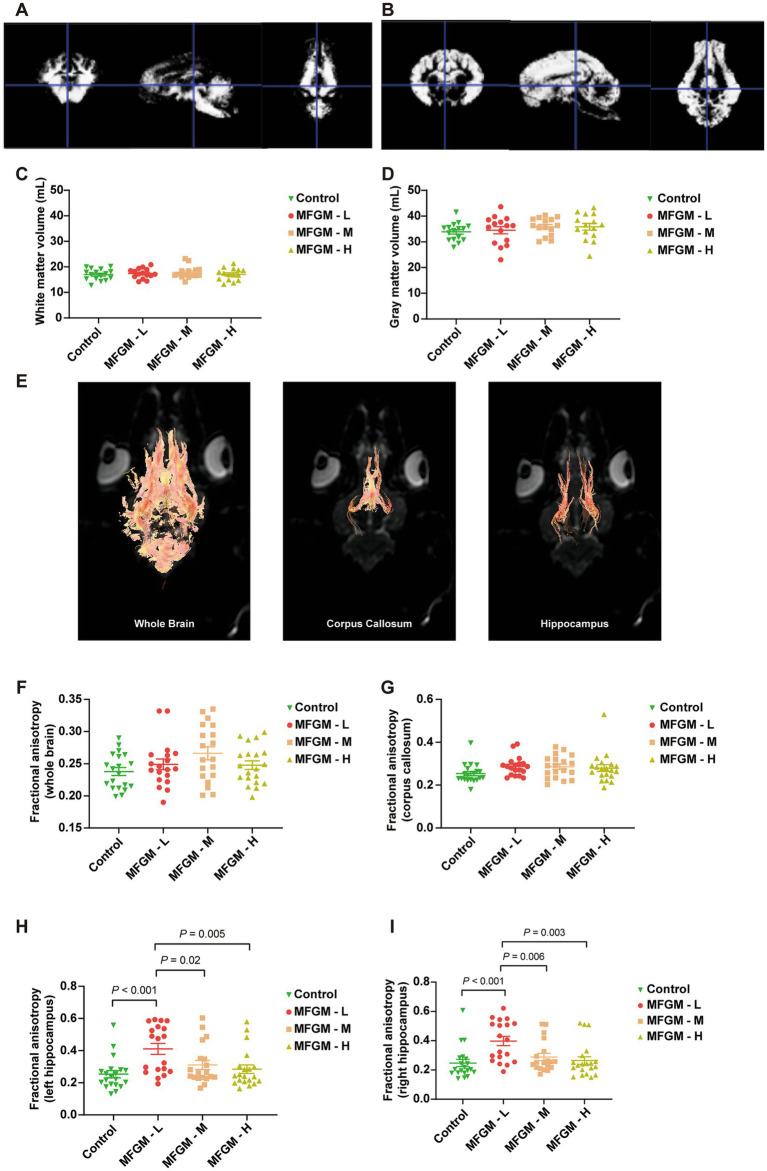
The volume and fractional anisotropy of white matter and gray matter determined by MRI. Representative images of white matter **(A)** and gray matter **(B)** determined by VBM. The volume of White matter **(C)**, Gray matter volume **(D)**, Representative image of Fractional anisotropy in of the whole brain, corpus callosum, and hippocampus **(E)**. Fractional anisotropy analysis of the whole brain **(F)**, corpus callosum **(G)**, left hippocampus **(H)**, and right hippocampus **(I)**. *n* = 20 in Control and MFGM-H groups; *n* = 19 in MFGM-L and MFGM-M groups. Data were presented as mean ± SEM.

On day 30, all the piglets were recruited for an MRI scan to measure the gray and white matter volume. The results showed that the representative white and gray matter images were based on VBM, the standard accepted test method (Figures 3A,B). There were no significant differences in white and gray matter volume calculated based on VBM [*F*_(3, 57)_ = 0.442, *p* = 0.724; F_(3, 57)_ = 0.760, *p* = 0.521, respectively; Figures 3C,D]. The other index of brain development was the trace of fiber bundles, with the representative images of fractional anisotropy (FA) determined by DTI in the whole brain, corpus callosum, and hippocampus shown in Figure 3E. No differences in FA were found among each group both in the whole brain [*F*_(3, 74)_ = 2.237, *p* = 0.091; Figure 3F] and corpus callosum [F_(3, 74)_ = 1.665, *p* = 0.182; Figure 3G]; However, there was a statistically significant difference in FA in the left hippocampus among the four groups [F_(3, 74)_ = 5.882, *p* = 0.001]. A Bonferroni post-hoc analysis revealed significant differences between Control and MFGM-L (*p* < 0.001), MFGM-M (*p* = 0.040), and MFGM-H (*p* = 0.005; Figure 3H). The same trend could be seen in the right hippocampus [F_(3, 74)_ = 6.269, *p* < 0.001]. A Bonferroni *post-hoc* analysis showed that MFGM-L fiber bundles were much better than control (*p* < 0.001), MFGM-M (*p* = 0.006), and MFGM-H (*p* = 0.003; Figure 3I). The MRI results were consistent with T-maze tests, indicating that MFGM-L contributed to memory-improving and white matter fiber growth.

### 3.4 The correct rate of the piglet was related to fa in the hippocampus

**Figure 4:**
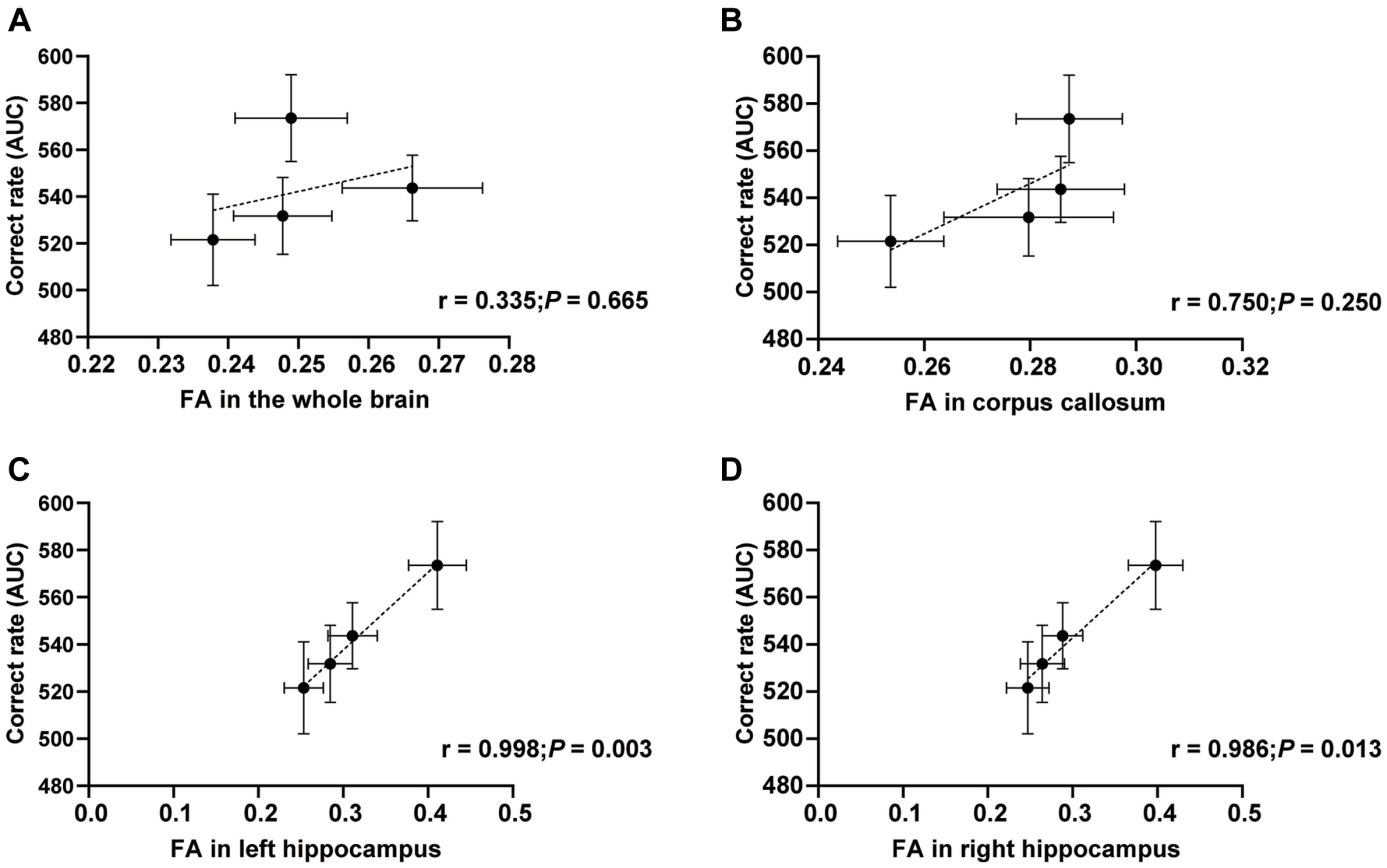
Correlations between FA and correct rate. Correlations between FA in the whole brain **(A)**, corpus callosum **(B)**, left hippocampus **(C)**, right hippocampus **(D)**, and correct rate (AUC). *n* = 20 in Control and MFGM-H groups; *n* = 19 in MFGM-L and MFGM-M groups. Data were presented as mean ± SEM.

We performed a correlation test to find the relation between FA and the correct rate in spatial T-maze tests. Specifically, the FA values of the whole brain, corpus callosum, left and right hippocamps white matter fiber bundles were correlated with the area under the curve (AUC) of the correct rate. The correlation between FA in the whole brain and correct rate (AUC) was no difference (*r* = 0.344; *p* = 0.665; Figure 4A). Similarly, the correlation between FA in the corpus callosum and correct rate (AUC) was also no statistical difference but more trend than the whole brain (*r* = 0.750; *p* = 0.250; Figure 4B). However, compared to the above results, the correlation between FA in the left hippocampus and correct rate (AUC) was another story. More correlation and statistical differences were observed (*r* = 0.998; *p* = 0.003; Figure 4C). Analogously, the right hippocampus also correlated with FA, and the correct rate was observed (*r* = 0.986; *p* = 0.013; Figure 4D). This figure shows that the FA value in the hippocampus correlates with the correct rate in the T-maze task.

### 3.5 The dose-dependent effect of BDNF expression in brain tissue

**Figure 5:**
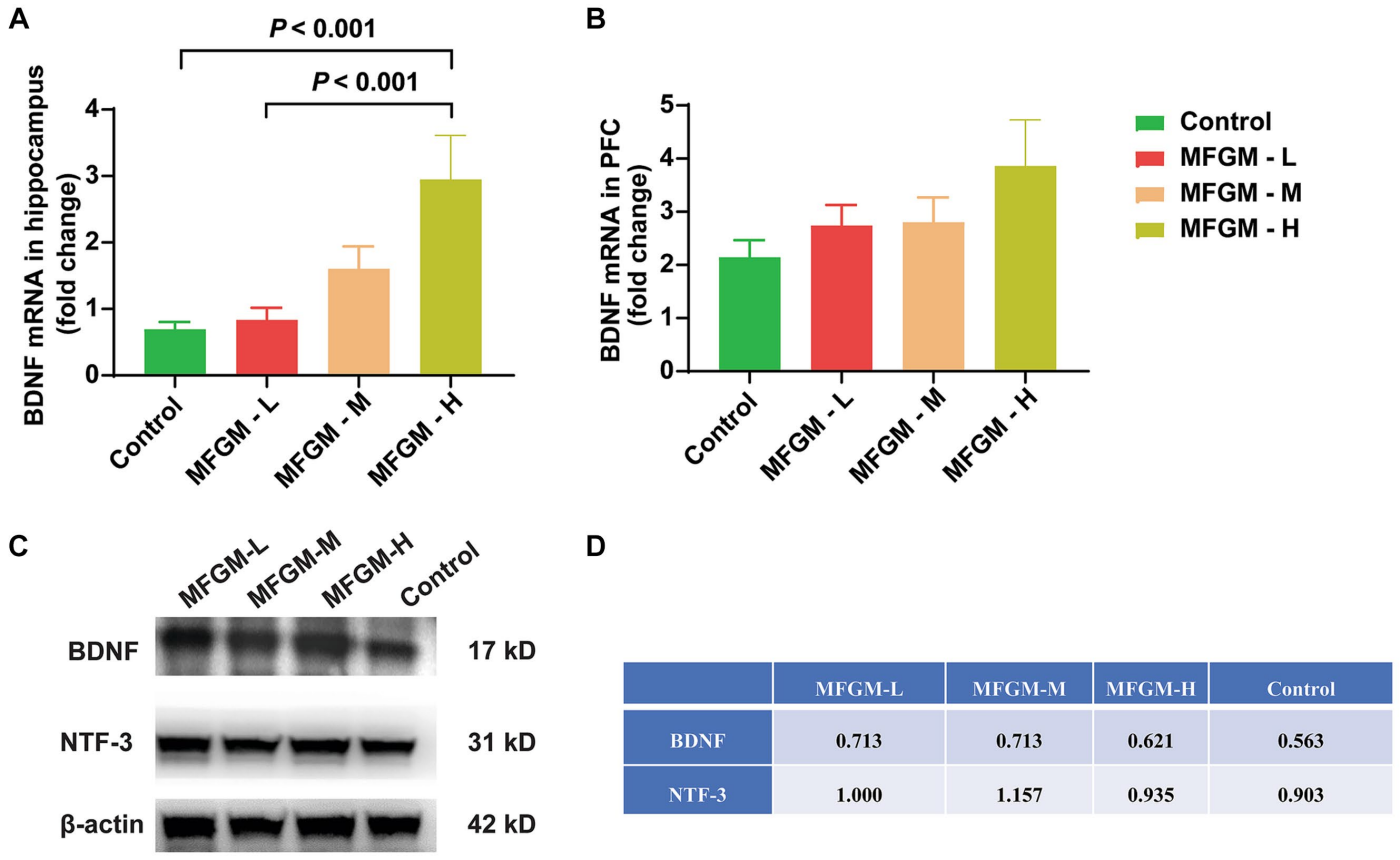
The relative expression of BDNF and NTF-3 in the brain. The expression of BDNF mRNA in the hippocampus **(A)** and Prefrontal cortex (PFC) **(B)** relative to β-actin. Control (*n* = 15), MFGM-L (*n* = 18), MFGM-M (*n* = 13), MFGM-H (*n* = 16); The protein expression of BDNF and NTF-3 in the hippocampus in each group **(C,D)**. Data were presented as mean ± SEM.

We further studied the mechanism of MFGM function by examining the expression of BDNF, which plays a role in the growth, maturation (differentiation), and maintenance of the neurons (21). Neurotrophin-3 (NT-3), including BDNF, nerve growth factor, and neurotrophin-4/5, play an important role in neurogenesis (22). The acquisition and consolidation of spatial memory depend on the correct functioning of both the hippocampus (23) and prefrontal cortex (24), where the highest levels of BDNF are found in the central nervous system (25), so we analyzed changes in BDNF in the hippocampus and PFC. There was a significant difference in relative BDNF mRNA expression in the hippocampus among each group [*F*_(3, 58)_ = 7.332, *p* < 0.001]. A Bonferroni post-hoc analysis showed that the BDNF mRNA expression was higher in MFGM-H than in control (*p* < 0.001) and MFGM-L (*p* < 0.001; Figure 5A), indicating that a high dose of MFGM promoted the expression of hippocampal BDNF and the development of the neurons. However, the expression of BDNF in PFC was no statistical difference but only a trend [*F*_(3, 70)_ = 1.590, *p* = 0.200; Figure 5B]. The protein expression of BDNF and NTF -3 was determined by Western blotting, mixing each sample of one group as a new sample. It showed that BDNF expression was the highest in MFGM-L and MFGM-M and lowest in control, while NTF-3 expression was the highest in MFGM-M and most lacking in control (Figures 5C,D). These results demonstrated that after the addition of MFGM, regardless of the dosage, the expression of BDNF and NTF-3 in brain tissues would increase.

## 4 Discussion

This study investigated the effect of various concentrations of MFGM-enriched diet on memory improvement and brain development in piglets. The concentration of MFGM did not show a dose-dependent relationship in spatial T-maze tests, and the 1.74 g MFGM per 100 g diet was the best choice, especially in memory-improving and white matter fiber bundle enhancement. This was confirmed for the first time through behavioral and imaging analysis.

Piglets were used in this study due to their similarities to infants in terms of brain structure, function, and digestive system function (26). During 30 days of suckling, piglets grow equivalent to an average six-month-old infant (16). Additionally, piglets are advantageous in short growth cycles, fast breeding, and low cost compared to other laboratory animals, such as nonhuman primates (27). The physiological superiority of nonhuman primates is undeniable; however, their limited availability, specialized management requirement, ethical considerations, and high costs significantly restrict their practical applications (28). Besides, the typical litter size of piglets can exceed 10, which is suitable for our large-scale experiments. Due to ethical considerations, exploring the effects of early life insults, specifically infection or nutritional deficiencies, on the development of the nervous system and cognitive abilities in human subjects is infeasible. Consequently, neonatal piglets offer an unparalleled translational animal model that can effectively tackle some of these concerns above.

The spatial T-maze test, widely recognized in the industry, assessed piglet memory. This choice was based on existing literature (29) and its ease of use. The test was designed to evaluate both “place” and “direction” learning, with piglets being trained to locate milk rewards consistently in a designated spot using visual cues (19). In our study, the piglets improved their ability to find the milk reward over time, as indicated by reduced latencies in making choices. By day 5 of acquisition, the piglets reached the criterion of 80% correct choices. However, when the location of the reward was reversed, the correct rate significantly dropped compared to the last acquisition day. Nonetheless, the correct rate gradually increased over 3 days in the reversal phase, which aligns with previous findings (11, 30). While the control group performed reasonably well in the T-maze test, there was still room for improvement, making it a sensitive measure of study and memory. We observed instances where piglets would only turn left or right in the T-maze test without relying on visual cues, resulting in a precise correct rate of 50%. These observations suggest that these piglets might be utilizing egocentric processing, which is self-centered and defined relative to the subject, a mechanism dependent on the striatum, as opposed to allocentric processing, which is world-centered and reliant on the hippocampus (19, 31). Notably, when we impaired spatial memory using the anticholinergic drug Scopolamine, the performance of piglets in the T-maze task sharply declined. This demonstrates the effectiveness of the T-maze task in evaluating learning and working memory (19).

MFGM supplementation did not affect the gray and white matter volume. Although total brain size is the most established neuroanatomical predictor of general intelligence, it only accounts for approximately 5% of the variation in individuals’ intelligence quotients (32). Early brain development depends on various functional factors, including energy metabolism, myelination, neurotransmission, and synaptic plasticity, which require further investigation (33).

Imaging analysis, the FA value based on DTI, was used to demonstrate the white matter fiber trace and represent brain development and neurodevelopment (34, 35). It was confirmed that DTI was a powerful tool for the study of early brain development, and higher FA was associated with maturational trajectories of primary and heteromodal association fibers (36). We found that changes in FA of the hippocampus in MFGM groups were much more significant than FA in the whole brain or corpus callosum. Mudd et al. confirmed that dietary sialyl lactose influenced brain development by increasing the diffusion tensor in the corpus callosum (37–39). We did not find the FA changes in the corpus callosum, which suggested that the corpus callosum and hippocampus may be differentially sensitive to MFGM supplementation (40, 41). The selection of cognitive domains – episodic memory, working memory, and information processing speed are contingent upon the pivotal role played by the hippocampus in memory consolidation and information processing. By administering a comprehensive battery of cognitive tests, Morris Moscovitch et al. explored potential links between alterations in hippocampal morphology and changes across multiple cognitive domains (42). Previous investigations into the hippocampus have attested to its heightened involvement in episodic, as opposed to other types of memory. Prior research has also demonstrated a positive association between higher FA level measurement in hippocampal volume and enhanced working memory and verbal memory (43). In accordance with previous literature, our study aligns with the anticipated outcomes.

The lack of a dose-dependent relationship for MFGM in the low-dose group may be attributed to the saturation of MFGM at low doses. Since phosphatidylcholine existed in the control diet, and control piglets did not perform poorly in the T-maze task, it was not easy to discover better T-maze results as the increasing of MFGM concentration in the diet. The inability of the organism to absorb higher doses of MFGM may result in rapid metabolism, thereby hindering the manifestation of a dose-dependent relationship. The MFGM dosage design is based on the effective dosage reported in previous literature and the content of MFGM in both domestic and foreign breast milk. We may reduce the MFGM concentration and redesign the MFGM dose gradient in the following studies to determine the optimal MFGM dose for brain development in neonatal piglets.

The mechanism of MFGM efficacy remains unclear, and previous research aimed to determine the influencing factors in brain development through the expression of BDNF and NTF-3, the important factors in neurodevelopment. In the present study, MFGM-H was found to perform the best in terms of BDNF mRNA expression in the hippocampus, while NTF-3 protein showed only a trend and no statistical difference. After excluding experimental error in PCR and WB, we still observed inconsistent levels of BDNF mRNA and protein expression across different groups. The disconnection might be due to the post-transcriptional modifications, such as mRNA splicing, processing, degradation, RNA editing, and RNA interference, which worth further investigation. It was observed that the positive impact of MFGM on BDNF mRNA enhancement in hippocampus was solely evident at high dosages. This result, however, contradicts the spatial T-maze accuracy outcomes. We discovered conflicting evidence regarding the relationship between BDNF and postnatal neuronal development upon conducting an in-depth investigation. For instance, decreased BDNF has been associated with reduced neuroplasticity, compromised neuronal health, and impaired recovery (44). Studies on BDNF knockout mice have demonstrated impulsive behavior, hyperactivity, and learning deficiencies (45). Conversely, another study suggests that dysregulation of BDNF contributes to the development of intellectual disability, and BDNF levels could serve as an early biomarker for identifying such disabilities (46). Yeom et al. have found a negative correlation between high peripheral BDNF levels and intelligence, behavioral problems, and intellectual disability in preschool children (47). In summary, BDNF at physiological levels supports learning and memory. However, both elevated and reduced levels of BDNF can disrupt inhibitory and excitatory neurotransmission in the brain, leading to a decline in synaptic refinement and memory impairment (48).

Moreover, it is essential to consider that BDNF is a downstream effector protein that works at the cellular level by modulating synaptic plasticity, promoting differentiation and survival of neurons (49, 50). Although BDNF levels only showed significant improvement in the high-dose group, the crucial indicator for improved neural development remains the enhancement of behavioral functions. Evidence suggests that the relationship between changes in behavioral processes and BDNF levels is non-linear. Therefore, future research will further investigate the pathways through which MFGM improves brain development (51). Thus, other factors beyond the presence or absence of MFGM may have played a role in BDNF expression.

Despite the lack of significant differences in our study, it is important to continue exploring the potential effects of MFGM on brain development and function, given its complex composition and its demonstrated benefits for enhancing the richness and orderliness of white matter fiber bundles and behavioral benefits such as improved decision-making and spatial discrimination (52). BDNF research should remain a vital aspect of this investigation, as it is essential for understanding the mechanisms underlying neural plasticity, learning, and memory formation (44). Further studies are necessary to elucidate the interaction between MFGM and BDNF, among other factors, and their impact on cognitive and behavioral outcomes (53). Ultimately, the findings from such research could inform the formulation of more optimized infant formulas that promote optimal brain development and improve long-term health outcomes.

This study presents certain limitations. Firstly, the MFGM concentration did not ascertain the ideal dosage for promoting brain development. It is suggested that the MFGM concentration should be systematically reduced for future investigations to determine the optimal dosage. Secondly, a wider range of biomarkers other than BDNF may be explored to monitor the progress of brain development. Moreover, it is crucial to account for changes in the blood–brain barrier in forthcoming studies.

Overall, this research highlights the dosage of MFGM in the diet, the need for further research in this area, and the potential impact of this research on infant nutrition and development. This study contributes to the market promotion of MFGM dairy products and the development of the Chinese dairy industry.

## 5 Conclusion

The study investigated the impact of diet with 1.74 g/100 g MFGM on memory improvement in piglets. The effect was possibly due to white matter connection and through the modulation of the BDNF-independent pathway.

## Data availability statement

The raw data supporting the conclusions of this article will be made available by the authors, without undue reservation.

## Ethics statement

The animal study was approved by Institutional Animal Care and Use Committee (IACUC) of West China Hospital Sichuan University. The study was conducted in accordance with the local legislation and institutional requirements.

## Author contributions

YZ, ZhZ, and TL conceived and designed the experiments. YZ, BZ, LG, QL, CT, and YW performed the animal experiments. ZP was responsible for imaging. XT was in charge of animal welfare. ZiZ, JH, SD, and SM-Y prepared reagents and materials. YZ, BZ, and ZZ wrote the manuscript. YY polished up the paper. All authors contributed to the article and approved the submitted version.
