# Supplementary material for: Milk fat globule membrane promotes brain development in piglets by enhancing the connection of white matter fiber trace
Source: Front Nutr. 2023 Nov 23;10:1248809. doi: 10.3389/fnut.2023.1248809 (PMC10701284; doi:10.3389/fnut.2023.1248809)
Supplement: Supplementary file 2 [file Data_Sheet_1.DOCX]

Supplementary figures


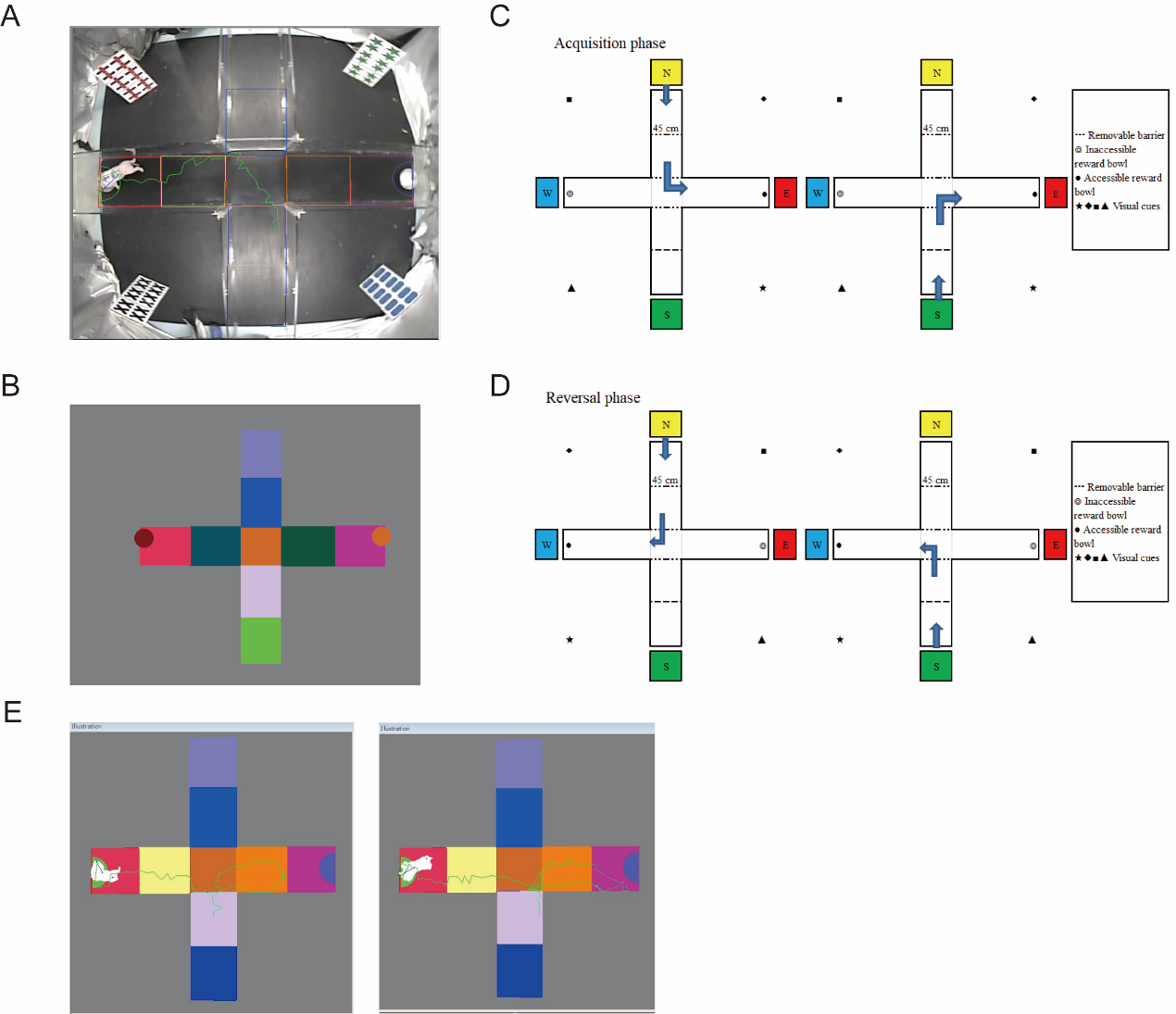


**Supplementary Figure 1** The design of spatial T-maze

Photograph of spatial T-maze used in cognitive behavioral experiments. Four different visual cues were placed in the corners of the maze. Piglets were trained to locate the reward milk using visual cues (A). The arena of spatial T-maze generated by Topscan software (B). The schematic pictures of spatial T-maze in the acquisition phase (C) and reversal phase (D). Blue arrows indicate the right route from N or S in different phases. (E) The trace of piglets walking and turning around in the maze. (W: west; E: east; N: north; S: south)


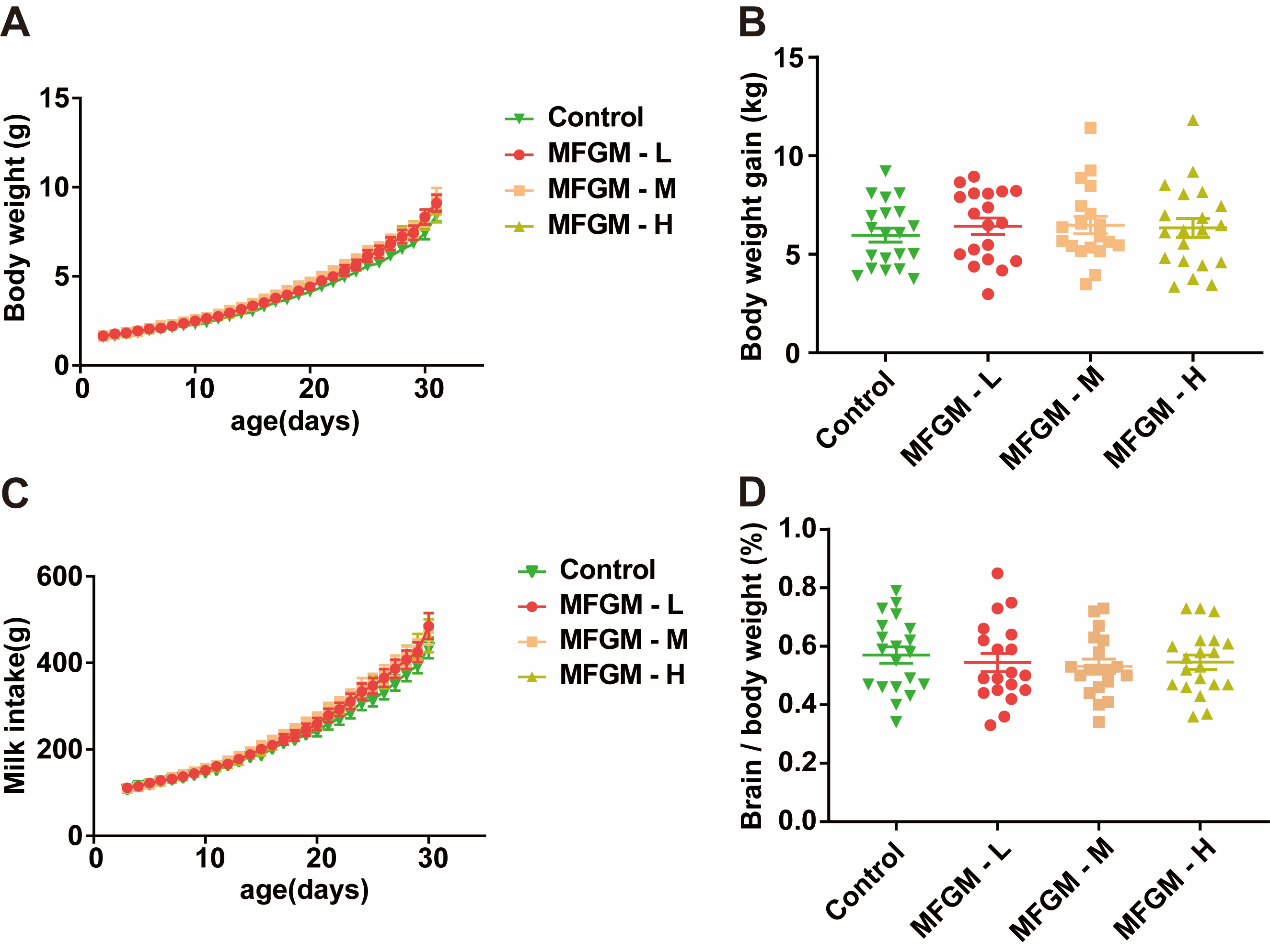


**Supplementary Figure 2** Piglet growing curve and milk intake index

Changes in body weight. piglets from postnatal days 2 to 31 (A). Body weight gain during 29 days (B). Changes in milk intake from postnatal days 2 to 30. (C); Brain/body weight rate in each group (D). Control (n = 19), low dose of MFGM-fed (MFGM-L, n = 19), medium dose of MFGM-fed (MFGM-M, n = 20), and high dose of MFGM-fed (MFGM-H, n = 20). Data were presented as mean ± SEM.
